# Cases of Hydrophobia; Not Arising from the Bite of a Rabid Animal

**Published:** 1825-11

**Authors:** 

**Affiliations:** Surgeon 2d Regiment of Guards.


**Art. III.**—*Cases of Hydrophobia; not arising from the Bite of a Rabid Animal.* By — Whymper, M.D. Surgeon 2d Regiment of Guards.

George Edgar, a florid and remarkably robust soldier of the Coldstream Guards, quartered at the Region-house near Dublin, had passed one of the hottest mornings of July, about a mile from the fort, angling. At noon he returned to his barrack to dinner, and whilst there discovered that some part of his fishing tackle had been left behind: he suspected that he should find it entangled by the weeds, and knowing that the tide was rising, he ran back with all possible speed; but, on his arrival, the spot at which he had fished was already covered by the sea: he instantly plunged into it, with a body bathed with perspiration, and remained in it for an hour and a half. Throughout the night he felt chilly, was sleepless and depressed. Towards the next morning, he complained of a sense of weight on the upper part of the thorax of the left side, relieved by pressure, and a benumbed aching, striking down the left arm, and most felt in the wrists. His countenance was observed to be pale, his lips livid; and his whole character was that of excitement, alarm, and restlessness. In this state he was sent into Dublin, to the hospital under my charge. The appearance of the man, on his admission there, was so peculiar, that, although he was complaining but little, and my usual visit was expected within half an hour, I was immediately summoned.

I was forcibly struck with this extreme anxiety of his countenance, and the irritability of his manner, strongly contrasted with his accustomed respectful and soldier-like demeanour, and generally healthy appearance. He complained of slight pain, but much oppression under the five upper ribs of the left side, with obtuse aching of the left arm and shoulder, which were weak and powerless. His pulse was oppressed and wiry, and somewhat irregular and intermittent; his breathing was slow and laboured, frequently interrupted by deep, sobbing sighs. His skin was rather cool, but he was tormented by excessive thirst: at the same time he resisted drinks, as "taking away his breath." His bowels were rather constipated.

I considered that the above symptoms indicated a loaded state of the large veins and right side of the heart. I directed a large bleeding, a warm bath, and free purging by oleum ricini. The blood coagulated slowly; the crassamentum did not contract. The pulse became regular; and he was so much relieved, that, throughout the remainder of the day, the symptoms were tranquillised.

On the following morning, I had the mortification to find him much worse. He had passed a restless night. He was freed from pain, but greatly irritated by the sense of weight and numbness above mentioned; from which he sought some relief by making the nurse forcibly press with her fist on the fourth and fifth ribs. His respiration had rapidly become, beyond expression, anxious: he breathed by continual low-drawn convulsive sobbings: his inspiration appeared solely the act of volition; for the slightest diversion of his attention from it interrupted it, and it was restored by a violent and convulsive struggle, such as is incidental to a sense of impending suffocation. His pulse was again irregular and intermittent; his look wild; his pupils dilated, yet exceedingly sensible to changes of light. He felt giddy, and all the senses were inordinately excited. His bowels had acted much. The skin was still cold, and covered by moisture; his thirst excessive, but he could not now bear the sight or sound of fluids without a sense of horror, and the wish that they should be instantly removed. The attempt to swallow, by interrupting respiration, induced the convulsions. He felt so persuaded that, if he could swallow, he should be cured, that he asked for a piece of bread, which he directed one of the attendants to form into a pellet, and cover with butter. In giving this to him, his thigh was accidentally touched: the unexpected sense of cold, caused even by the touch of the butter, made him dart from the bed to the floor in horrid convulsions. He could not swallow it. In another instance the nurse, in crossing the ward, spilt a little water: the sound induced convulsion. The distant shutting of a door,—the touching any part of his person unexpectedly,—the moving of the window-blinds by the air, so as to admit a sudden flickering of light,—the agitation of the room, by people moving quickly near the bed,—in short, any circumstances which produced a sudden or unexpected impression on any one external sense, was sufficient to induce convulsion; and that, as it seemed to me, chiefly by withdrawing his attention from the business of breathing.

As he had been lulled by the former bleeding, &c. I determined to repeat it; but the sight of the blood, or the sound of its fall into the basin, brought on the paroxysm. He became faint after losing eight ounces, and for a time quiet. I now attempted to give him a large dose of opium and hyoscyamus; but, after having the pills in his mouth, he could not be persuaded to try to swallow them. A large blister was placed over the lower cervical and upper dorsal vertebræ, and another over the left upper ribs.

Whilst the faintness continued, comparative relief continued; and he even succeeded in getting down a little warm tea, but at the cost of an effort and strangulation which had nearly destroyed him, and from which he recovered with a pulse small, rapid, and tremulous; a skin bathed by cold sweat; an inexpressible anxiety, and horror of all the excitements above mentioned. He kept constantly upright in his bed, moving his position, and his pillows and bedclothes, every second; declaring that, if he could relieve his throat and chest from the load which choaked him, he should be well. He was continually spitting, hawking, and gnashing his teeth; and, whilst his appearance was so appalling that his attendants shrunk from him with dread and astonishment, his mind was lucid, and free from any intention of mischief.

This scene of suffering continued for about thirty hours, when a state of rapid collapse succeeded. The nervous excitability suddenly ceased; the respiration became natural; the pulse, though scarcely perceptible, regular; he swallowed whatever was given for his support with ease. In his mind he was so calm, that he knew that death was approaching; had prayers read to him; and sunk into a syncope, from which he never recovered.

The symptoms of the case will be allowed, I suppose, to be very similar to those of the hydrophobia, "ex morsu animalis rabidi;" for there was most remarkably the *cujuslibet potionis*

*fastidium et horror;* and so fearful was the gnashing of his teeth during the continued effort to clear his mouth and throat from the viscid saliva which he thought to be choking him, during the latter stages of the disease, that his attendants, without any suspicion of hydrophobia, scarcely dared to approach him; and, without any forced analogy, an apparent *cupiditas mordendi* may be assumed to have been present.

It is hardly necessary to recapitulate the various other symptoms above enumerated, which will not fail to strike the attention of those who have had the misfortune to see the disease as it follows the bite of a rabid animal. The most careful inquiry denied the suspicion of the disease in question having had any such cause: and, till the instant of the violent shock given to the nervous system by the sudden and long-continued application of cold to this man's person when extremely heated, he was in perfect health.

If I am right in concluding, as I do, that this case was similar in all respects, excepting its cause, to those which are consequent to the admission of the rabid poison into the system, I may be permitted to doubt the correctness with which Cullen classes hydrophobia as a disease of the natural functions; and nosological distinctions drawn from causes are often fallacious. To fix the seat of this disease, would be the most favourable step towards its cure. Various authorities have of late, from the frequent appearance of inflammation of the mucous membrane of the pharynx, &c. inferred that the proximate cause of this disease is the inflammation of this part, inducing the spasmodic disturbance of its own and neighbouring muscles. But this state of membranous inflammation is by no means an invariable concomitant even of hydrophobic paroxysms: dissection of those who have died from this disease, whether excited or not by the rabid poison, has often shown this; and believing, as I do, that the cases proceeding from that poison are extremely rare, but quite similar to hydrophobia following other causes, I am tempted to think that every cause which can produce it has its first and chief action on the nerves of the vital functions, disturbing the equilibrium of influence between the nerves which regulate the voluntary and involuntary processes of respiration. "The nerves of respiration," says Mr. Bell, "so peculiar in relation and function, are differently influenced by disease from the other divisions of the nervous system: their functions are left entire when the voluntary nerves have ceased to act, and they are sometimes strangely disordered whilst the mind is entire in all its offices, and the voluntary operations perfect. In hydrophobia, the respiratory system is affected: hence the convulsions of the throat, the paroxysms of suffocation, the speechless agony, whilst the voluntary motions are free."

In the above case, the remarkably flaccid and extenuated state of the parietes of the heart, torn as easily as soaked pasteboard,—the unusual dryness of the serous surfaces of the thorax and pericardium,—and the distention of the great veins and right auricle, were the only remarkable morbid phenomena found on dissection; and, were I to venture upon a *ratio symptomatum* of the above case, or of the various cases characterised as hydrophobia which have come to my knowledge, it appears to me that I could, most satisfactorily to myself, derive it from the primary disturbance of the nerves governing the great vital muscles of the circulation and respiration.

I subjoin the following case, as a curious consequence of the first; and I submit that it also corroborates my idea of the seat of such diseases. The sufferer was an attendant upon Edgar.

William Cooper was admitted into the hospital early in the morning, and stated that he had been tolerably well the day before, but had felt low, and depressed, and weak for some days: "he knew of no reason for this, excepting that he had not felt quite well since he saw Edgar's sufferings." He complained of having been attacked, within a few hours of his admission, by a considerable pain in the left side of the chest, in the region of the heart. He had a disposition to hiccup; an urgent sense of impending suffocation, from which he obtained momentary relief by frequent, deep, and spasmodic inspiration; constant effort to clear his fauces and trachea from viscid mucus; urgent thirst, but disliked the attempt to drink, as "it took away his breath," and induced strangulation. Skin cool and clammy; pulse small, wiry, and irregular. He felt relieved by lying and pressing on the side affected. His countenance was peculiarly distressed and anxious; mind alarmed, and evidently occupied by the similarity of his situation to that of Edgar,—which indeed was sufficiently striking; psillismus; natural functions regular, except the great increase of the secretion of limpid urine.

11 A.M.—He took immediately a drastic purge, and was bled *ad deliquium*.

3 P.M.—No severe return of spasm for one hour after the bleeding. He was now free from any severe pain, but complains much of oppression, and singular hesitation of speech; pulse very irregular, and intermitting; bowels freely open: rejects drinks.—A strong purging draught ordered every four hours.

On the following day, the symptoms of disturbance of the respiration and circulation, as detailed above, had severely returned. Respiration very rapid, requiring more attention; and spasms more easily excited by slight external stimuli. He was again bled *ad deliquium*.

From this period he gradually recovered, and was dismissed from the hospital to the country in about a fortnight. But his health has received a severe shock; he was again in hospital, during the whole of the last winter, with pulmonic disorder. He has still a remarkable agitation of manner and hesitation of speech, and is about to be discharged from the service of the regiment as an invalid. The pulmonic disease under which he now labours is constant dyspnœa, palpitation, with occasional attacks of asthmatic paroxysms. There has been no evidence either of tubercles or chronic inflammation. The close connexion of the recurrent nerve with the plexuses of the heart and lungs, seem to explain sufficiently the affection of the muscles of speech.

